# Enrichment of c-Met^+^ tumorigenic stromal cells of giant cell tumor of bone and targeting by cabozantinib

**DOI:** 10.1038/cddis.2014.440

**Published:** 2014-10-16

**Authors:** L Liu, E Aleksandrowicz, P Fan, F Schönsiegel, Y Zhang, H Sähr, J Gladkich, J Mattern, D Depeweg, B Lehner, J Fellenberg, I Herr

**Affiliations:** 1 Department of Molecular OncoSurgery, General, Visceral and Transplantation Surgery, University of Heidelberg and German Cancer Research Center (DKFZ), Heidelberg, Germany; 2 Department of Experimental Orthopedics, Orthopedic University Hospital, Heidelberg, Germany

## Abstract

Giant cell tumor of bone (GCTB) is a very rare tumor entity, which is little examined owing to the lack of established cell lines and mouse models and the restriction of available primary cell lines. The stromal cells of GCTB have been made responsible for the aggressive growth and metastasis, emphasizing the presence of a cancer stem cell population. To identify and target such tumor-initiating cells, stromal cells were isolated from eight freshly resected GCTB tissues. Tumorigenic properties were examined by colony and spheroid formation, differentiation, migration, MTT (3-(4,5-dimethylthiazol-2-yl)-2,5-diphenyltetrazolium bromide) assay, immunohistochemistry, antibody protein array, Alu *in situ* hybridization, FACS analysis and xenotransplantation into fertilized chicken eggs and mice. A sub-population of the neoplastic stromal cells formed spheroids and colonies, differentiated to osteoblasts, migrated to wounded regions and expressed the metastasis marker CXC-chemokine receptor type 4, indicating self-renewal, invasion and differentiation potential. Compared with adherent-growing cells, markers for pluripotency, stemness and cancer progression, including the CSC surface marker c-Met, were enhanced in spheroidal cells. This c-Met-enriched sub-population formed xenograft tumors in fertilized chicken eggs and mice. Cabozantinib, an inhibitor of c-Met in phase II trials, eliminated CSC features with a higher therapeutic effect than standard chemotherapy. This study identifies a c-Met^+^ tumorigenic sub-population within stromal GCTB cells and suggests the c-Met inhibitor cabozantinib as a new therapeutic option for targeted elimination of unresectable or recurrent GCTB.

Giant cell tumor of bone (GCTB) is a very rare, osteolytic neoplasm deemed histologically benign, but it is locally aggressive and destroys bone and overlying soft tissue.^1,2^ Surgery has been the preferred treatment for GCTB; however, the lesion tends to recur locally. In ~6% of cases, the development of lung metastases has been observed.^3, 4, 5^ GCTB has a predilection for the epiphyseal/metaphyseal region of long bones and the spine and thus can cause substantial morbidity.^6^ For patients with unresectable GCTB, the use of chemotherapeutics, bisphosphonates, radiation, radiofrequency thermal ablation and arterial embolization are palliative options with limited effects on tumor control.^7, 8, 9^ Recently, denosumab, a RANKL inhibitor, has been approved for GCTB, and it targets, especially the neoplastic stromal cells, which express high concentrations of RANKL.^9,10^

GCTB is composed of three different cell types: multinucleated, osteoclast-like giant cells, CD68^+^ phagocytic histiocytes and fibroblast-like stromal cells. The stromal cells have been identified as the neoplastic cell population,^11, 12, 13^ and it is believed that they develop from mesenchymal stem cells (MSCs).^14,15^ The latter notion is supported by studies that demonstrate involvement of MSCs in tumor development—for example, in the development of sarcoma.^16^

According to the hypothesis, cancer stem cells (CSCs) are responsible for growth, invasion, metastasis and therapy resistance of cancer, because this small sub-population within the tumor mass is thought to survive conventional cytotoxic therapy because of activated defense and survival mechanisms.^17^ CSCs are characterized by self-renewal potential and the ability to differentiate, thereby generating a heterogeneous cell population of the originating tumor.^18, 19, 20^ In addition, CSCs are proposed to mediate uncontrolled growth, therapy resistance, invasion and metastasis.^21^ Markers for CSCs have been identified in various tumor entities, and the selected marker-positive fractions can reconstitute the original tumor in immunodeficient mice.^22^ There are several surface markers for CSCs of different tumor entities and the c-Met marker represents such a typical CSC sub-population.^23, 24, 25^

c-Met belongs to the group of receptor tyrosine kinases and has a key role in cell survival, growth, angiogenesis and metastasis.^26^ c-Met and its physiologic ligand hepatocyte growth factor (HGF) are required for normal mammalian development and have an important role in epithelial–mesenchymal interactions during organ morphogenesis.^26^ The intracellular signaling cascades activated by c-Met include the RAS-MAPK and PI3K-AKT pathways, as well as NF-*κ*B and Wnt/GSK-3*β*/*β*-catenin signaling.^26^ Many carcinomas overexpress c-Met, and the surrounding stroma overexpresses HGF. Currently, the therapeutic potential of the c-Met inhibitor cabozantinib (XL184) is intensively investigated. Cabozantinib is a potent dual inhibitor of c-Met and VEGFR-2 signaling.^25,27^ The clinical efficacy of cabozantinib in several progressed tumor entities is under investigation in randomized phase II studies.^28^ At the end of 2012, cabozantinib (Cometriq) was approved by the FDA for the treatment of patients with progressive medullary thyroid carcinoma.^29^ Cabozantinib shows promise in preventing prostate cancer spread to bone because tumors were reduced on bone scans, and bone pain decreased after patients received cabozantinib.^30^ These data may be of importance for GCTB, but until now, cabozantinib has not been investigated for the treatment of primary bone tumors.

In the present study, we demonstrate that a c-Met^+^ sub-population of low-passage stromal cells isolated from eight freshly resected GCTB specimens possess self-renewal, differentiation and migratory potential, as well as the ability to form tumors *in vivo*. By comparing attached-growing c-Met^low^ and spheroidal c-Met^high^ cultures, we identified enhanced pluripotency, stemness and progression, as well as the enrichment of a c-Met^+^ population. Most importantly, cabozantinib strongly inhibited the self-renewal potential and *in vivo* growth of GCTB stromal cells. Thus, cabozantinib may be considered an effective future therapeutic option for the targeted elimination of a tumorigenic stromal sub-population in non-resectable or recurrent GCTB.

## Results

### GCTB stromal cells exhibit CSC features

**Figure 1:**
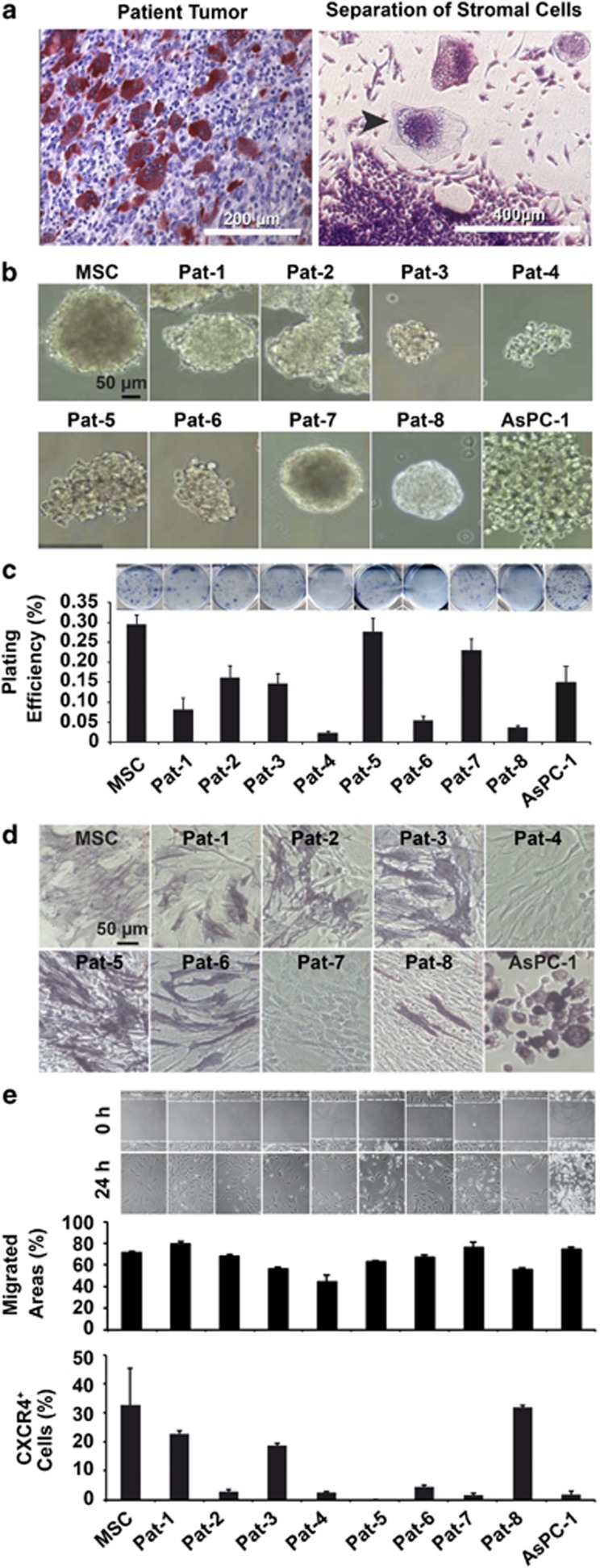
GCTB stromal cells exhibit self-renewal activity*.* (**a**, left) Representative paraffin section out of 20 of a surgically resected GCTB specimen after TRAP staining at × 200 magnification. (Right) Osteoclast-like giant cells and stromal cells in culture after digestion of the tumor tissue. The arrow marks a giant cell surrounded by stromal cells at × 400 magnification. (**b**) GCTB stromal cells isolated from eight different patient tumors (Pat-1 to Pat-8) were seeded at clonal density in low-adhesion plates. The spheroids were grown until day 7 and photographed at × 100 magnification. MSCs or established pancreatic cancer cells (AsPC-1) served as controls. Data are representative of three independent experiments with similar results. (**c**) Colony-forming assay of cells plated in medium containing 10% FCS at clonal density of 200 cells/well. Cells were grown without change of medium for 2 weeks, followed by evaluation of fixed and Coomassie blue-stained colonies consisting of at least 50 cells. The plating efficiency as a percentage was calculated using the following formula: 100 × number of colonies/number of seeded cells. Data are presented as the mean of two experiments performed in sextuplicate (*n*=12)±S.D. (**d**) The differentiation potential was examined after incubation of cells in osteogenic differentiation medium for 10 days. Alkaline phosphatase expression was detected using BCIP/NBT. Cells were evaluated under × 200 magnification using a Nikon Eclipse TS100 microscope (Nikon Corporation, Sendai, Japan). Data are representative of three independent experiments with similar results. (**e**) Cells were cultured to 90% confluence before the cell layer was scratched with the tip of a pipette. Closure of the wounded region was evaluated 24 h after scratching by microscopy at × 100 magnification (pictures upper part). CXCR4 expression of GCTB-derived stromal cells was quantified by FACS analysis. Fluorescence intensities±S.D. of eight GCTB stromal patient-derived specimens and controls are shown (diagram lower part). Data are representative of three independent experiments with similar results. Data are representative of three independent experiments with similar results

Tartrate-resistant acid phosphatase (TRAP) staining of paraffin sections shows the typical GCTB histology, including a large amount of TRAP^+^, red giant cells surrounded by TRAP^−^ stromal cells and histiocytes (Figure 1a). Because histiocytes and giant cells do not survive in cell culture, and the stromal cells are thought to drive tumor growth,^11, 12, 13^ we separated stromal cells from eight freshly resected tissues of GCTB (Supplementary Table S1). To evaluate the tumor-initiating potential, we performed sphere and colony-forming assays. All patient-derived specimens were able to grow independently of anchorage as spheres 7 days after seeding in a serum-free medium were supplemented with growth factors (Figure 1b). The morphology of the different cell populations was heterogeneous, because anchorage-independent cultured cells were round shaped and formed multicellular clustered spheroids. In contrast, the cells in adhesive plates were spindle shaped with a fibroblastic-like morphology. Likewise, all cell lines formed colonies 2 weeks after seeding at clonal density (Figure 1c), a finding that was similar to that observed in MSC and AsPC-1 cells, which served as positive controls. The capacity for sphere formation was highest in Pat-1, Pat-2, Pat-5, Pat-7 and Pat-8 cells, and the capacity for colony formation was highest in Pat-5 and Pat-7 cells, followed by Pat-1, Pat-2 and Pat-3 cells, whereas Pat-1 and Pat-4 cells had a very low colony-forming potential. For evaluation of the differentiation potential, we primed the cells for osteogenic differentiation and detected the presence of osteoblast-like cells by BCIP/NBT staining of alkaline phosphatase. Pat-2, Pat-3 and Pat-5 cells were highly positive for alkaline phosphatase, Pat-1, Pat-6 and Pat-8 cells expressed intermediate levels of alkaline phosphatase and Pat-4 and Pat-7 cells were negative for alkaline phosphatase compared with MSC and AsPC-1 cells, which served as positive controls (Figure 1d). To analyze the migration potential, the cells of each line were grown to 90% confluence, before a scratch was made into the cell layer. Twenty-four hours later, cells that migrated to the wounded region were detected by microscopy (Figure 1e, upper part). Compared with the positive controls, all GCTB stromal cells exhibited migration potential, which was highest in Pat-1, Pat-3 and Pat-7 cells because the wounded region was completely closed. These findings suggest the presence of invasion potential, and we strengthened this conclusion by examining the expression of the CXC-chemokine receptor type 4 (CXCR4), which is a marker for invasion potential and CSCs.^24^ Staining of the cells with a CXCR4-specific antibody and FITC-labeled secondary antibody followed by FACS analysis of positive cells revealed the highest CXCR4 expression in Pat-1, Pat-3 and Pat-8 cells, whereas all other patient-derived specimens had an intermediate expression of CXCR4, except for Pat-5 cells, in which CXCR4 expression was not detectable (Figure 1e, lower part). Taken together, all of the eight examined GCTB stromal cells possess features associated with CSCs, but there are cell line-specific differences, which may be because of patient-specific heterogeneity in GCTB. According to the analyzed parameters, Pat-1 cells had the highest CSC potential, followed by Pat-2, Pat-3 and Pat-7 cells, whereas Pat-6 and Pat-8 had intermediate CSC features, and Pat-4 cells had the lowest CSC potential (Supplementary Table S2).

### Expression of markers for pluripotency and tumorigenesis in GCTB stromal cells

**Figure 2:**
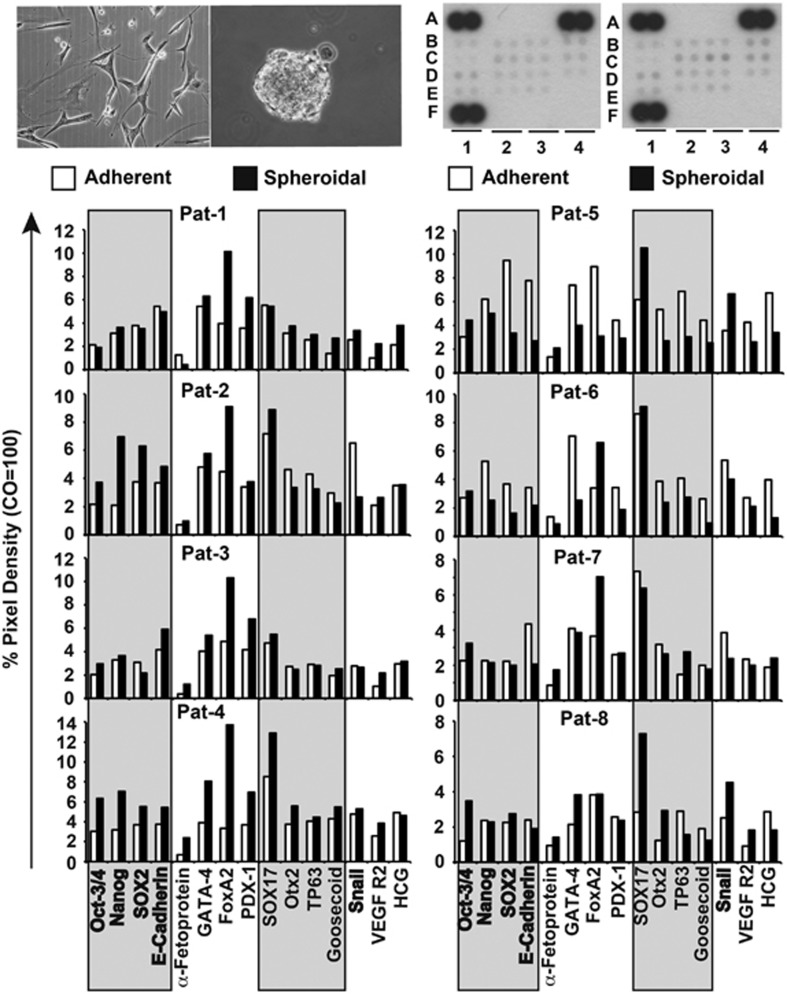
GCTB stromal cells express stem cell markers. Proteins from adherent- or spheroidal-growing cells (upper left pictures) were prepared and incubated with the nitrocellulose membranes of an antibody array kit for the detection of human pluripotent stem cell markers. The binding of proteins to antibodies spotted on the membrane was detected using biotinylated secondary antibodies, streptavidin-HRP and chemiluminescence (upper right pictures). The pixel density was quantified using ImageJ software and normalized to the mean pixel intensity of reference spots located at the coordinates A1, A4 and F1 on the membrane. Spot E4 is the negative control, where PBS instead of the antibody was spotted onto the membrane. This experiment was performed with eight different primary cell lines once in duplicate for a general overview and the mean values are shown

To further elucidate the existence of a stem cell population in GCTB, we performed an antibody protein array, which detects the levels of 15 protein markers associated with pluripotency and tumorigenicity (summarized in Supplementary Table S3). Based on recent publications, which demonstrate that the more aggressive tumor cells can be selected by anchorage-independent culture in a serum-free, growth factor-supplemented medium,^25,31,32^ we used these culture conditions and compared the marker expression in adherent- and spheroidal-growing stromal cells of all eight GCTB specimens (Figure 2). We found that a basal expression of most of the markers were already in adherent cultures—for example, OCT3/4, Nanog, SOX2 (essential for self-renewal and pluripotency) or E-cadherin and Snail (Snail is involved in the inhibition of E-cadherin during EMT), suggesting that a basal potential for pluripotency is present in stromal cells of GCTB. However, the expression of these proteins was further enhanced by anchorage-independent culture in the majority of cell lines, suggesting that the spheroidal growth leads to an enrichment of a more aggressive sub-population. Although the pattern of induction was not consistent between the primary cell lines, this is not necessarily a weakness or limitation, but reflects the heterogeneous differences between GCTB specimens of different patients. Patient-derived specimens with the highest levels of activation were Pat-1, Pat-2, Pat-3, Pat-4 and Pat-8 cells, whereas Pat-7 cells showed an intermediate induction (Supplementary Table S4). Pat-5 and Pat-6 cells showed inhibition of marker expression in spheroidal cultures, but they exhibited significant basal expression of stem cell-related factors already in adherent-growing cultures. Taken together, these results suggest that signaling factors important for pluripotency and tumorigenesis are expressed in adherent-growing GCTB stromal cells, and a population with high expression could be further induced in six of the eight examined patient-derived specimens by anchorage-independent spheroidal culture.

### GCTB stromal cells form xenograft tumors in fertilized chicken eggs

**Figure 3:**
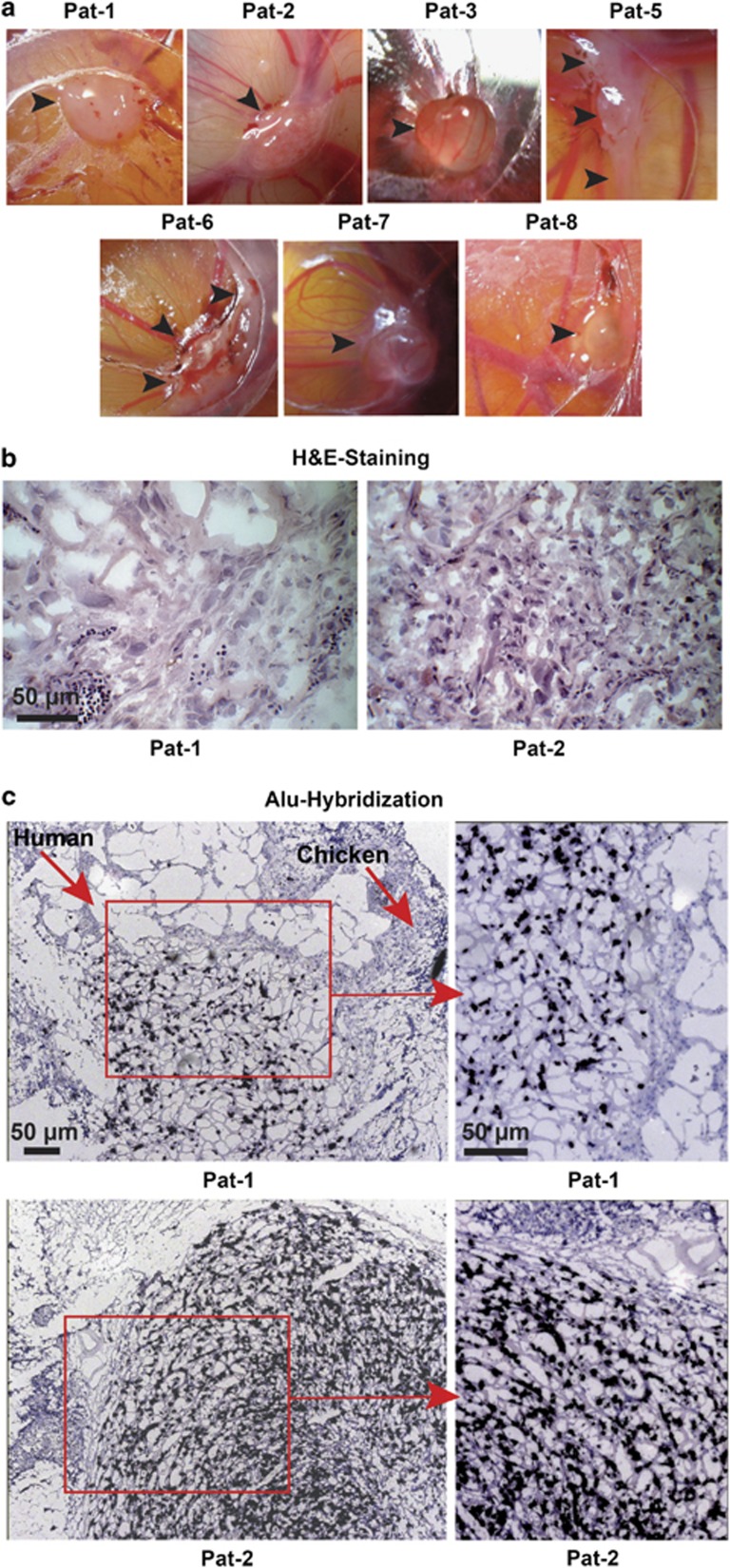
GCTB stromal cells form tumor xenografts. (**a**) GCTB stromal cells (1 × 10^6^) derived from seven different patients were transplanted on the CAM of fertilized chicken eggs (*n*=8 per cell line) at embryonal development day 10 and photographed at day 17. The arrows mark the tumor xenografts. (**b**) H&E staining of representative frozen xenograft sections derived from Pat-1 and Pat-2 cells. (**c**) Alu hybridization of egg xenograft tissue derived from Pat-1 and Pat-2 cells. Dark blue-labeled cells of human origin and unlabeled chicken cells are marked by arrows. Pictures were taken at × 400 magnification, and the bar indicates 50 *μ*m

For evaluation of tumor xenograft formation, we transplanted the adherent-growing cultures on the chorioallantoic membrane (CAM) of fertilized chicken eggs, because this method has been recently described as a promising *in vivo* model for examination of GCTB.^33^ We assume that fertilized chicken eggs may be suited for selection of a CSC population, although this is not been described so far. However, according to the CSC hypothesis, the ability to reconstitute a tumor in immunodeficient mice is suggested as a major characteristic of a CSC.^22^ Chicken embryos do not have a developed immune system,^34^ and therefore may be a suited alternative to the conventional immunodeficient mouse model. After transplantation, all of the patient-derived specimens, except the very slowly growing Pat-4 cells, formed cartilage-like, poorly perfused, glass-white tumor xenografts upon transplantation of 1 × 10^6^ cells (Figure 3a). H&E staining of tissue sections revealed porous tissue, consisting of larger human cells and invading smaller chicken cells, as exemplified for xenograft tissue from Pat-1 and Pat-2 cells (Figure 3b). Hybridization of the xenograft tissue with a probe specific for human Alu sequences ensured that most of the cells in the xenograft tumors were of human origin (Figure 3c). Taken together, the ability of a sub-population of the stromal cell fraction of GCTB to grow *in vivo* suggests a tumorigenic potential of these particular sub-populations.

### c-Met is expressed on the cell surface of a putative CSC sub-population of stromal cells of GCTB

**Figure 4:**
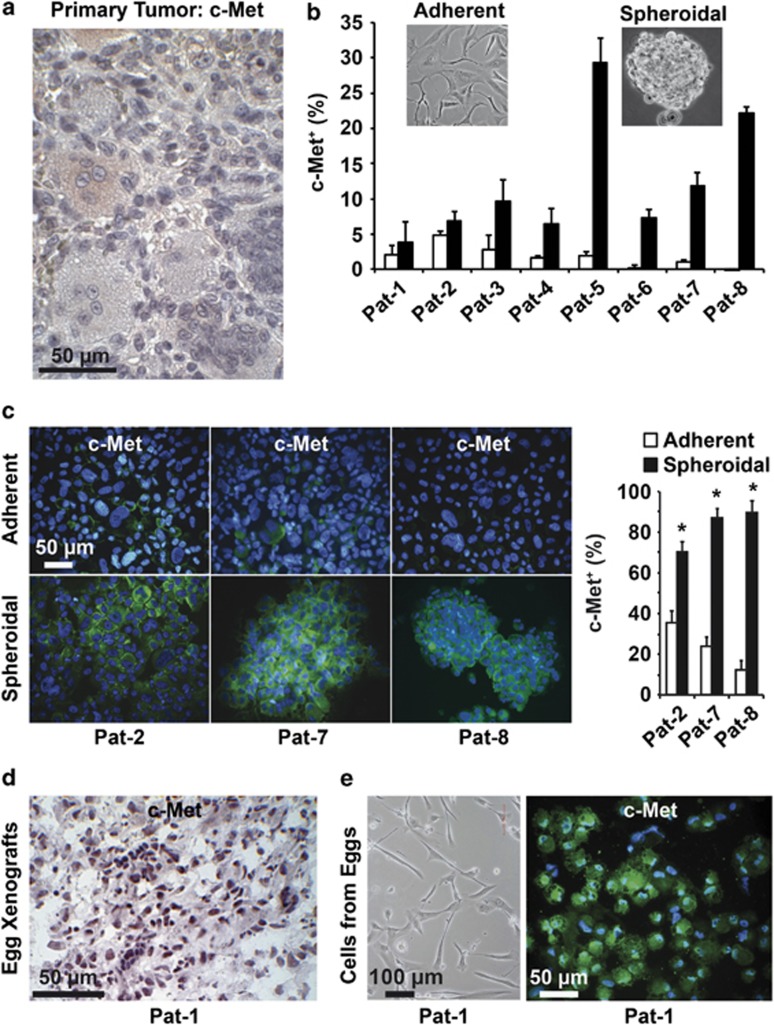
c-Met is enriched in spheroidal cultures and xenografts. (**a**) Representative c-Met staining (light red) of a section of surgically resected GCTB tissue at × 200 magnification (*n*=5). (**b**) Flow cytometry analysis of c-Met expression in adherent- (white bars) and spheroidal- (black bars) growing GCTB stromal cells. (**c**) Cytospins were performed from adherent- and spheroidal-growing GCTB cells derived from Pat-2, Pat-7 and Pat-8, followed by immunofluorescence staining of c-Met (green) and DAPI counterstaining of cell nuclei (blue), followed by fluorescence microscopy at × 400 magnification. The bar indicates 50 *μ*m. c-Met^+^ and c-Met^−^ cells of 10 vision fields were evaluated and are presented as the percentage of c-Met^+^ cells on the right. (**d**) Sections from egg xenografts (*n*=8) derived from stromal cells of Pat-1 were stained with c-Met antibody, and positive cells were detected by immunohistochemistry (dark violet). (**e**) Characterization of adherent-growing cells isolated from egg xenografts (*n*=8) from Pat-1. Representative photograph (*n*=3) of the cell morphology (left) and that of c-Met^+^ cells after immunofluorescence staining (right: green) with DAPI counterstaining (blue) at × 400 magnification. The scale bars indicate 100 and 50 *μ*m, respectively. The data shown in (**b** and **c**) are representative of three experiments performed in duplicates (*n*=6)

To define a surface marker for tumorigenic stromal GCTB cells, we focused on c-Met, which has been identified as a strong CSC surface marker in other tumor entities, for example, pancreatic ductal adenocarcinoma.^25^ Immunohistochemistry of patient-derived GCTB tissue sections revealed that the cells are almost negative for c-Met, although some giant cells and some stromal cells exhibited a slightly enhanced red positive staining. We evaluated the percentage of c-Met^+^ stromal cells by counting 10 vision fields of five different tissue sections of GCTB and found that 1.78±0.8% c-Met^+^ stromal cells (Figure 4a). This indicates a weak basal c-Met expression in accordance with the CSC theory, which states that not all, but only a small sub-population within the tumor mass has tumorigenic potential.^22^ Because our former results suggest that a tumorigenic population is enriched in spheroidal cultures, we compared the expression of c-Met in adherent- and anchorage-independent growing stromal cells of the eight GCTB-derived specimens by FACS analysis. In adherent cultures, a very low basal c-Met expression could be detected (Figure 4b). However, 7 days after spheroidal culture, the expression of c-Met increased in all specimens, with the most significant levels in Pat-3, Pat-4, Pat-5, Pat-6, Pat-7 and Pat-8 cells. These data were confirmed by c-Met immunohistochemistry and immunofluorescence staining of c-Met in adherent- and spheroidal-growing cells from Pat-2, Pat-7 and Pat-8 cells, and evaluation of the percentage of c-Met^+^ cells (Figure 4c and Supplementary Figure S1). To examine whether the *in vivo* growth in eggs may also lead to enrichment of a c-Met^+^ sub-population, we performed immunohistochemistry from xenografts derived from chicken eggs. Indeed, a high percentage of c-Met^+^ cells were detected, and a representative image of xenograft tissue from Pat-1 cells is shown (Figure 4d). This finding was confirmed by isolation of cells from the egg xenograft and c-Met immunofluorescence staining of the *in vitro* cultured cells (Figure 4e). Excitingly, the morphology of xenograft-derived cells was identical to the parental stromal cells, suggesting that the c-Met^+^ stromal cell sub-population is enriched by anchorage-independent culture and transplantation to chicken eggs.

### Spheroidal, but not adherent, GCTB stromal cells form c-Met^+^ xenografts in mice

**Figure 5:**
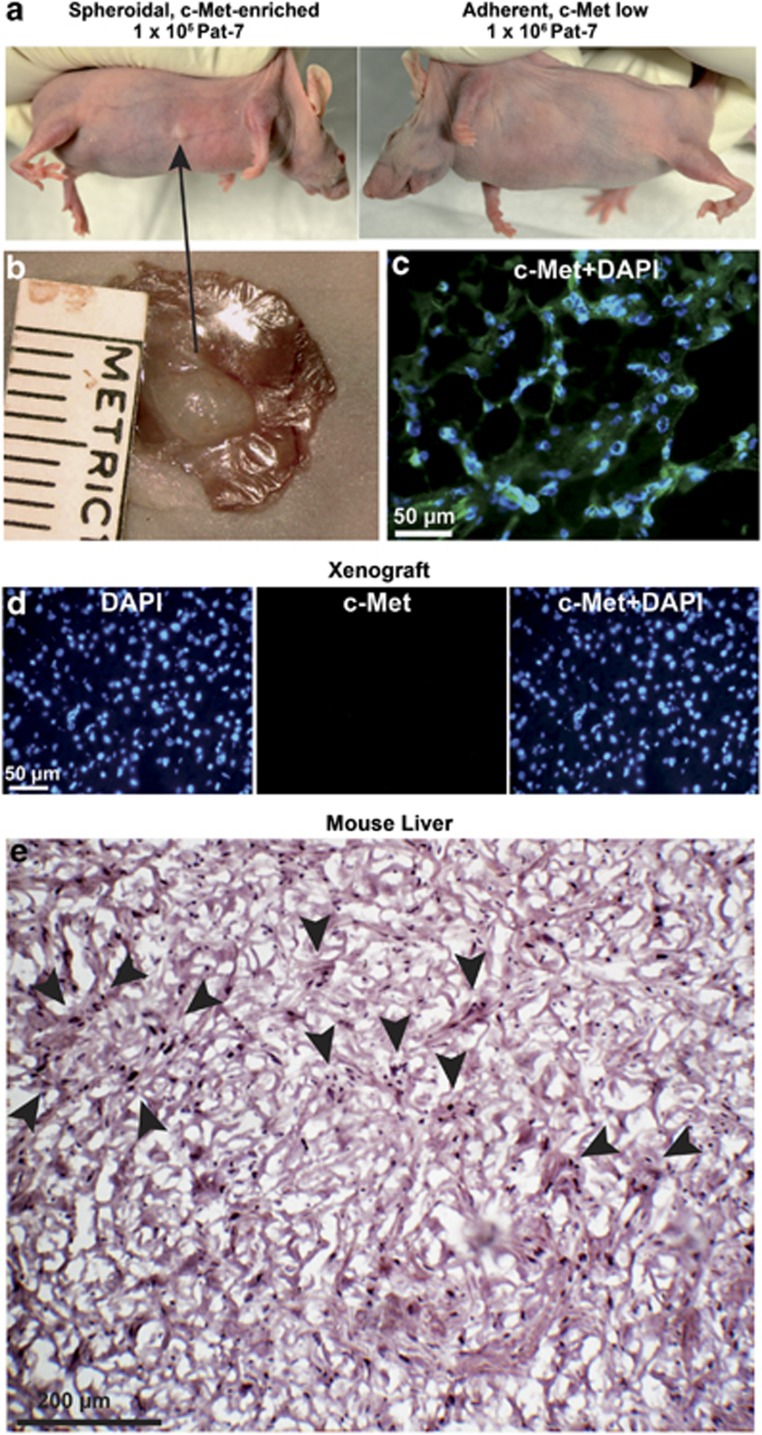
c-Met^+^ GCTB stromal cells form tumors in mice. (**a**) A total of 1 × 10^5^ spheroidal-growing and 1 × 10^6^ adherent-growing Pat-2, Pat-7 and Pat-8 cells were subcutaneously transplanted into the right and left flanks of 15 mice per cell line. Four months later, one mouse developed a tumor at the right flank at the site where spheroidal cells from Pat-7 cells were injected. (**b**) The tumor xenograft was resected, and the size of 5 × 3 mm^2^ was determined by calipers. (**c**) c-Met (green) staining merged with DAPI counterstaining (blue) of a frozen mouse xenograft section derived from Pat-7 cells. The scale bar indicates 50 *μ*m. (**d**) c-Met control staining (no signal) and DAPI staining (blue) of mouse liver sections (*n*=5). The scale bar indicates 50 *μ*m. (**e**) H&E staining of a frozen mouse xenograft section derived from Pat-7 cells. The scale bar indicates 200 *μ*m. Arrows indicate giant-like cells. Owing to a lack of tumor tissue, we could not further evaluate the features of the xenograft

To detect if the c-Met^+^ fraction only will form a tumor xenograft tumor in immunodeficient mice, which is described as a major CSC characteristic,^35^ 1 × 10^5^ c-Met^high^ spheroidal and 1 × 10^6^ cells c-Met^low^ adherent Pat-2, Pat-7 and Pat-8 cells were transplanted to the left flank and right flanks of 15 mice per cell line, respectively (Figure 5a). Four months after injection, the growth of a small cartilage-like tumor xenograft was observed in one mouse at the site of injection of Pat-7 spheroids (Figure 5b). The tumor cells were highly c-Met^+^ as evaluated by immunofluorescence staining and fluorescence microcopy (Figure 5c). In contrast, staining of mouse liver tissue with this antibody was negative (Figure 5d), suggesting that the c-Met^+^ tumor xenograft is of human origin. Furthermore, H&E staining suggests the presence of stromal cells surrounded by structures of scattered, giant-like cells (Figure 5e). This morphology resembles that of a GCTB patient tumor—although it is not identical. These exciting results suggest that a c-Met^+^ sub-population of stromal cells drives the tumorigenic growth of GCTB. The week yield and the very slow proliferation rate (4 months until a tiny tumor xenograft occurred) reflect the low malignant progression potential of GCTB.

### Targeting of c-Met^+^ stromal cells by cabozantinib eliminates CSC characteristics

**Figure 6:**
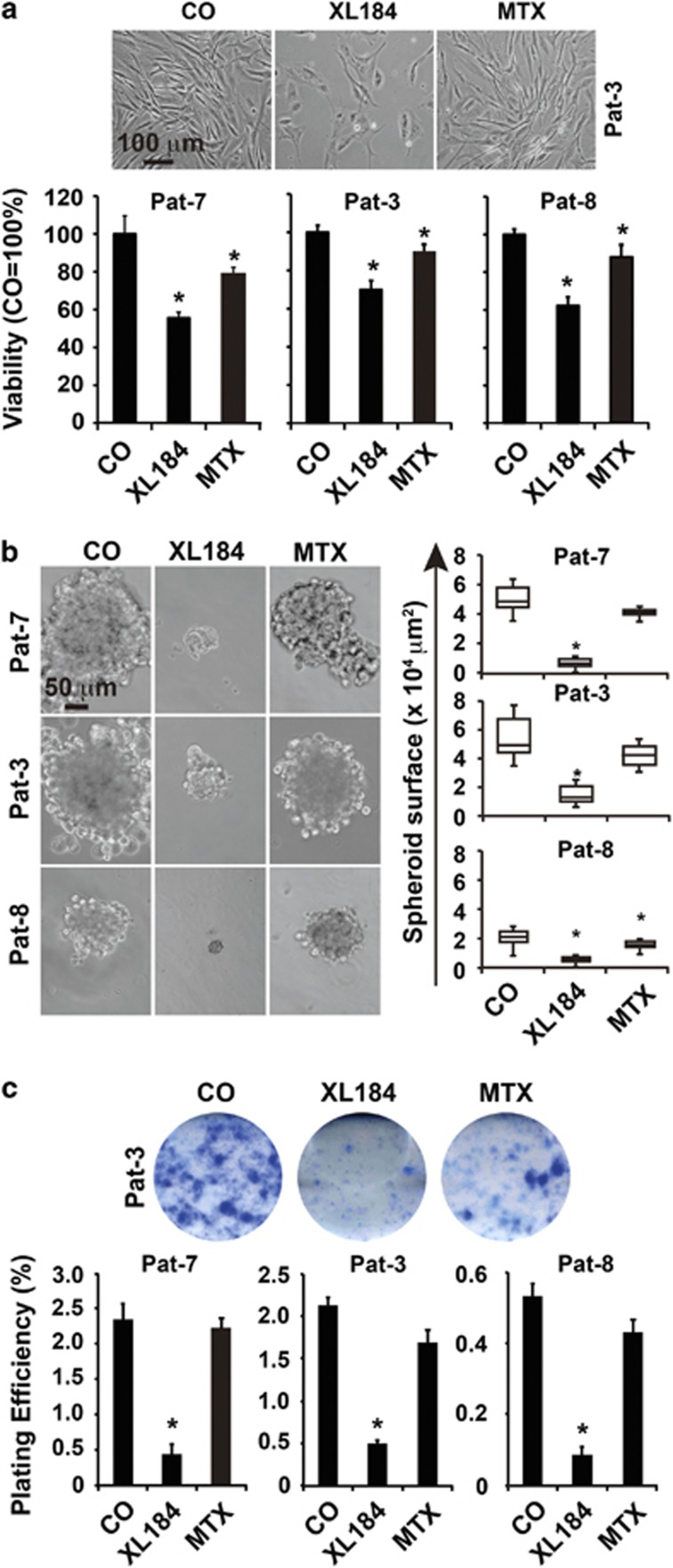
Cabozantinib reduces viability and spheroid and colony formation of GCTB stromal cells. (**a**) Adherent-growing GCTB stromal cells derived from three different patients were left untreated (CO) or were treated with cabozantinib (10 *μ*M, XL184) or methotrexate (100 *μ*M, MTX). Seventy-two hours later, the viability was measured by the MTT assay, and the control was set to 100%. (**b**) Spheroidal cultures were established as described in Figure 1b. After spheroid formation, the cells were left untreated or were treated as described above. Seven days later, spheroids were photographed, and the number and volume of spheroids (spheroid surface) were determined. (**c**) Cells were seeded at a density of 1.5 × 10^5^ cells/ml in six-well plates. After 24 h, the cells were treated as described above. Seventy-two hours later, cells were trypsinized, and 2000 viable cells of each group were seeded per well of a six-well plate. Colony formation was evaluated as described in Figure 1c. Representative pictures of colonies derived from Pat-3 are shown. The data shown are the mean±S.D. (*P<0.05; **P<0.01). Data are representative of three independent experiments with similar results. Data from (**a**) and (**b**) are representative of three experiments performed in triplicate (*n*=9) and data from (**c**) were performed two times in sextuplicates (*n*=12)

**Figure 7:**
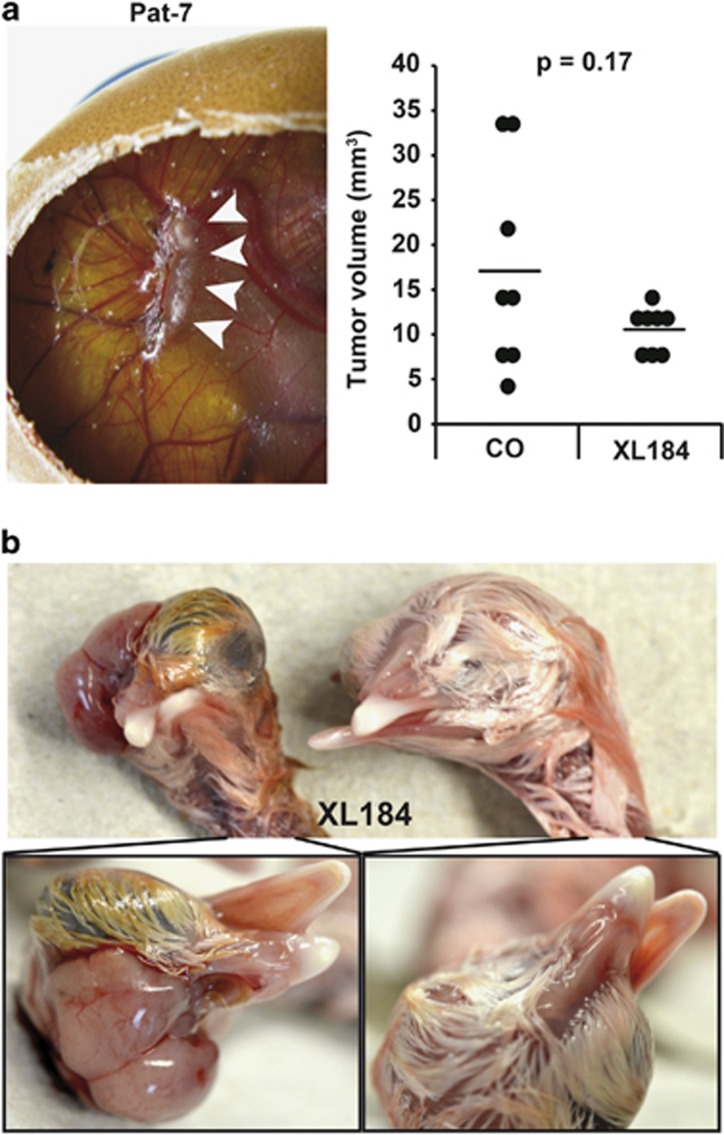
Cabozantinib reduces the tumorigenicity of GCTB stromal cells *in vivo*. (**a**) GCTB stromal cells (Pat-3, 1 × 10^6^/group) were left untreated or were treated with cabozantinib (10 *μ*M) *in vitro.* Twenty-four hours later, equal amounts of viable cells were transplanted in Matrigel on the CAM of fertilized chicken eggs at day 9 of embryonic development (*n*=8 per group). At day 17, the xenograft tumors were resected (representative picture of a xenograft tumor in a chicken egg), and the volumes were determined. The single data points and means of both groups are shown in the diagram on the left. (**b**) *In ovo* application of XL184 (10 *μ*M) to a 1-cm^2^ Whatman paper on the CAM until saturation at day 11 of embryonic development led to craniofacial malformation in two of seven chicken embryos as photographed at day 18 of embryonal development. In contrast, chicken embryos from untreated eggs developed normally

To further highlight the role of c-Met signaling in the survival of GCTB-derived stromal cells, we treated cells from three different patients with the c-Met inhibitor cabozantinib (XL184) and compared the effect to treatment with the cytotoxic drug methotrexate. Cabozantinib significantly reduced the cell number and viability, whereas methotrexate was minimally effective as evident from morphologic changes and the 3-(4,5-dimethylthiazol-2-yl)-2,5-diphenyltetrazolium bromide (MTT) assay (Figure 6a). This result was expected, because stromal cells of GCTB are considered to be chemotherapy resistant.^9^ To investigate whether cabozantinib affects the self-renewal potential, spheroidal-growing cells were left untreated or were treated with cabozantinib and methotrexate, respectively. Seven days later, the spheroids were photographed, and their volumes were determined (Figure 6b). Whereas cabozantinib nearly completely eliminated the spheroids, methotrexate was minimally effective. Similar results were obtained by examination of the self-renewal potential using the colony-forming assay (Figure 6c)*. In vivo*, pretreatment of stromal cells with cabozantinib, followed by transplantation on the CAM of fertilized chicken eggs, strongly reduced the tumor growth compared with untreated control cells (Figure 7a). Remarkably, two of seven embryos derived from eggs transplanted with cabozantinib-pretreated cells had craniofacial defects, which were not observed in embryos derived from eggs transplanted with untreated GCTB stromal cells (Figure 7b). Such developmental defects of chicken embryos are a rather rare event, and we did not yet observe it during transplantation experiments with other tumor entities and more than 5000 eggs in the meanwhile. These results suggest that the minimal residual concentrations of cabozantinib delivered by the pretreated and washed cells have interfered with c-Met-dependent signaling pathways, which are not only active in CSCs but also in embryonal development,^36^ thus underlying our suggestion that c-Met-mediated stem cell signaling is responsible for the tumorigenicity of a sub-population of GCTB stromal cells.

## Discussion

In the present study, we examined whether a tumorigenic sub-population may be present among stromal GCTB cells to define a specific targeting strategy. Although recent clinical studies suggest denosumab, a RANKL inhibitor, as a new strategy to target especially the neoplastic, RANKL-expressing stromal cells,^9,10^ this strategy does not necessarily target the most aggressive, tumorigenic sub-population of stromal cells. In our approach, we aimed to identify such a CSC-like stromal sub-population and to establish a therapeutic strategy for selective elimination. Our data show that a sub-population with enhanced expression of c-Met and other markers for tumorigenicity could be enriched by anchorage-independent cell culture that has been shown recently to favor the growth of CSCs in other tumor entities, whereas the more differentiated cells are deleted with time.^25,31,32^

Our study adds important information to the growing evidence that a CSC population is present within the stromal fraction of GCTB. Recently, Lan and co-workers.^37^ suggested that a Stro-1^+^ cell population within the stromal cells of GCTB possesses stem-like features. The authors of that study focused on Stro-1 because this is the best-known MSC marker that has been used to identify stem-like cells in some normal mesenchymal tissues.^37,38^ Additionally, Stro-1 was recently associated with CSCs of osteosarcoma.^39^ The *in vitro* studies by Lan *et al.*^40^ show that the FACS-sorted Stro^+^ population possesses a higher proliferation rate, self-renewal potential, multipotency and increased resistance toward cisplatin compared with the Stro^−^ population.^40^ Furthermore, the Stro-1^+^ stromal cells were CD44^+^, suggesting that CD44 may be another CSC marker of GCTB, although this is not yet examined in detail. To test whether c-Met^+^ cells are already present in patient tumors, or could be selected by *in vitro* culture, we stained tissue sections and detected a weak c-Met signal in some giant and stromal cells. This observation is in accordance with the CSC hypothesis, which suggests that only a small sub-population within the tumor mass has tumorigenic properties.^22^ In addition, the expression of c-Met may also occur in non-malignant cells, because c-Met is known to mediate cell mobilization of human MSCs, tissue repair and wound healing.^41^

Although our data support the notion that the stromal cells of GCTB represent the tumorigenic cell population,^11, 12, 13^ a role of polyploid giant cells in tumor progression and metastasis cannot be completely ruled out. In their recent work, Balke *et al.*^33^ transplanted fresh tumor tissue obtained from 10 patients with GCTB on the CAM of fertilized chicken eggs to generate a short-term model for GCTB. In accordance with our data, all 10 tissues grew on the eggs and were comprised of stromal cells, histiocytes and multinucleated giant cells. Although the histiocytes and giant cells were less numerous and the giant cells contained fewer nuclei than in the context of the primary patient tumor. These data support once more that the tumorigenic population is present within the stromal cell fraction of GCTB, because under *in vitro* and *in vivo* growth conditions, the giant cells are diluted with time. However, a function of giant cells in tumorigenicity is supported by recent findings in ovarian cancer in which giant cells were just now identified.^42^ These giant cells expressed normal and CSC markers and they divided asymmetrically and cycled slowly. They differentiated into adipose, cartilage and bone. A single giant cell formed cancer spheroids *in vitro* and generated tumors in immunodeficient mice. The giant cell-derived tumors gained a mesenchymal phenotype with increased expression of the CSC markers CD44 and CD133, and they were resistant to treatment with cisplatin.

Remarkably, our xenograft tumor obtained from transplantation of spheroidal-growing GCTB cells into mice contained giant cell-like structures embedded in a matrix of stromal cells. Therefore, it is tempting to speculate that the tumorigenic population of stromal cells is able to differentiate into giant cells. However, the low recovery rate obtained reflects the minor malignant potential of GCTB in patients. Our results are consistent with previous studies in which GFP-labeled GCTB stromal cells were injected into the tibia of immunodeficient Balb/c nu/nu mice.^43^ That study demonstrated the presence of green fluorescent, single stromal cells 1 year after injection. The latter scenario supports the notion and possibility that GCTB stromal cells survive in mice for a long time without forming large tumors. These results suggest that GCTB stromal cells remain in a dormant state *in vivo* and a yet unknown stimulus may lead to tumor progression and metastasis as observed in the clinical situation.

We tested the effect of the c-Met inhibitor cabozantinib as a new therapeutic option for the treatment of unresectable GCTB and demonstrated that cabozantinib prevents the self-renewal potential of stromal cells owing to the inhibition of anchorage-independent spheroidal growth and colony formation. Most importantly, the *in vivo* growth of cabozantinib-pretreated stromal cells in fertilized chicken eggs was inhibited, although cabozantinib induced severe developmental defects in chicken embryos. This obviously teratogenic effect of cabozantinib underscores the targeting of CSC signaling, because stem cell and developmental signaling is strongly related.^36^ Most importantly, cabozantinib has already been clinically used for the successful treatment of prostate cancer bone metastasis, efficiently reduced metastasis in bone scans and decreased bone pain.^30^ These clinical data together with our results suggest the clinical evaluation of cabozantinib as a new therapeutic option to target the tumorigenic c-Met^+^ stromal population of GCTB.

In conclusion, the present study identifies a c-Met^+^ tumorigenic sub-population within stromal GCTB cells and suggests the c-Met inhibitor cabozantinib as a potential novel treatment option for GCTB, especially in unresectable or recurrent cases.

## Materials and Methods

### Isolation of stromal cells from patient tissue of GCTB

Eight low-passage stromal patient-derived specimens of GCTB (Pat-1 to Pat-8) were obtained from different patients after resection of GCTB tissues at our clinic. The stromal cell population was isolated as described recently,^44^ resulting in a pure stromal cell population deleted of giant cells and histiocytes. Patient material was obtained under the approval of the ethical committee of the University of Heidelberg after written informed consent of the patients. The diagnoses were established by conventional clinical and histologic criteria according to the World Health Organization (WHO). All surgical resections were indicated by the principles and practice of oncologic therapy.

### Isolation of MSCs from the bone marrow of patients

MSCs were isolated from fresh bone marrow samples derived from the iliac crest of patients as described previously.^44^ Patient material was obtained under the approval of the ethical committee of the University of Heidelberg as described above.

### Established cell lines

The established human AsPC-1 pancreatic cancer cell line was obtained from the American Type Culture Collection (Manassas, VA, USA). Cells were authenticated throughout the culture by typical morphology and growth characteristics. To maintain the authenticity of the cell lines, frozen stocks were prepared from initial stocks. Subsequently, every 3 months, a new frozen stock was used for the experiments. Mycoplasma-negative cultures were ensured by monthly testing. Cells were cultured in DMEM (PAA, Pasching, Austria) supplemented with 10% heat-inactivated FCS (Sigma, Deisenhoffen, Germany) and 25 mmol/l HEPES (PAA).

### Treatment of cells

Cabozantinib (XL184, 99% Selleckchem, Houston, TX, USA) was dissolved in DMSO to produce a 20-mM stock solution. Methotrexate (Sigma) was diluted in 1 ml of 0.1 N NaOH/4 ml PBS to produce a 100-mM stock solution. The final concentrations of the solvents in the medium were 0.1% or less.

### Viability assay

Viability was measured using MTT as described previously.^45^

### Colony-forming assay

Cells were seeded in complete medium in 6-well tissue culture plates (TPP), and colony-forming assays were performed as described previously.^45^

### Spheroid assay

For formation of spheroids, cells were cultured in NeuroCult NS-A basal serum-free medium (human) (StemCell Technologies, Vancouver, BC, Canada) supplemented with 2 *μ*g/ml heparin (StemCell Technologies), 20 ng/ml hEGF (R&D Systems, Wiesbaden-Nordenstadt, Germany), 10 ng/ml hFGF-b (PeproTech, Hamburg, Germany) and NeuroCult NS-A Proliferation Supplement (StemCell Technologies). Cells were seeded at low densities (5 × 10^2^–2 × 10^3^ cells/ml, 1 ml/well) in 12-well low-adhesion plates (Corning Incorporated, New York, NY, USA). For quantification of the spheroid surfaces, the computer program ImageJ was used.

### Scratch assay

Cells (6 × 10^5^) were seeded in 6-well plates and grown to confluence overnight. A line was then scraped within confluent cells using the fine end of 10-*μ*l pipette tips (time 0). Images of migrating cells were sequentially acquired at 0 and 24 h. For quantification of the migrated area, the computer program ImageJ was used.

### Osteogenic differentiation

For osteogenic differentiation, cells were seeded at a density of 4.5 × 10^4^ cells/well in 6-well cell culture plates in ready-to-use NH OsteoDiff medium (Miltenyi Biotec, Bergisch Gladbach, Germany). Ten days later, alkaline phosphatase expressed by osteoblasts was visualized using FAST BCIP/NBT (Sigma), which produces a deep blue color change.

### Detection of c-Met and CXCR4 expression by flow cytometry

The cells (1 × 10^6^) were incubated with Venimmune (Aventis-Behring, Marburg, Germany) at 4 °C for 20 min to inhibit nonspecific binding of antibodies. After washing with PBS/5% FSC, the cells were incubated with FITC-conjugated rat mAb anti-human c-Met (eBioscience, Frankfurt, Germany) or with unconjugated rabbit mAb anti-human CXCR4 (Abcam, Cambridge, UK). After washing, the cells were used directly for FACS analysis (c-Met) or were incubated with FITC-labeled secondary Abs to detect unconjugated CXCR4 Ab. Fluorescence was examined using a Guava EasyCyte flow cytometer (Merck Millipore, Darmstadt, Germany). Gating was implemented based on negative control staining profiles.

### Immunofluorescence detection of c-Met

According to a standard protocol, 6-*μ*m frozen xenograft tissue sections were fixed in ice-cold acetone, followed by incubation with primary rabbit polyclonal Ab against human c-Met (Abcam). The nuclei were stained with DAPI (4,6-diamidino-2′-phenylindol; 1 *μ*g/ml). The secondary Ab was green fluorescent goat anti-rabbit Alexa Fluor 488 IgG (Invitrogen, Camarillo, CA, USA). The omission of the primary Ab served as a negative control. The signal was detected at × 400 magnification using a Leica DMRB fluorescence microscope (Leica, Wetzlar, Germany). Images of representative fields were captured using a SPOT^M^ FLEX 15.2 64-Mp shifting pixel digital color camera (Diagnostic Instruments Inc., Sterling Heights, MI, USA) and analyzed with SPOT Basic/Advanced 4.6 software (Diagnostic Instruments Inc.).

### Immunohistochemistry staining

Red staining of TRAP of GCTB tissue sections was performed as described in our recent publication.^46^ For SOX2 and Oct-4 staining, formalin-fixed, paraffin-embedded GCTB tissue sections were de-paraffinized in Roti-Histol (Carl Roth GmbH, Karlsruhe, Germany) and rehydrated in isopropanol. Antigen retrieval was performed using Dako target retrieval buffer pH 6 (Dako, Hamburg, Germany). The mouse mAbs against SOX2 (Merck Millipore) and OCT4 (Santa Cruz, Heidelberg, Germany) were used as primary antibodies. NBT/BCIP was used as a chromogen. For c-Met staining of GCTB patient tissue or GCTB-derived stromal cell xenografts, endogenous biotin was blocked using the Avidin/Biotin Blocking Kit (Vector, Burlingame, CA, USA) according to the manufacturer's instructions. Endogenous peroxidase was blocked by 0.3% H_2_O_2_ in methanol. A rabbit pAb against human c-Met (Abcam) was used as the primary antibody. Biotinylated goat anti-rabbit IgG (Vector) was used as the secondary Ab. The signal was amplified using the ABC Elite Kit (Vector). AEC was used as a chromogen. Samples were counterstained with hematoxylin (Mayer, Sigma-Aldrich, Steinheim, Germany) and mounted using Pro Tags Aqua mount (Quartett, Berlin, Germany). Omission of the primary Ab served as a negative control.

### Alu *in situ* hybridization

A digoxigenin-labeled probe for the human-specific Alu repetitive sequence was prepared by PCR. The following primers were used: ALU-F, 5′-CGAGGCGGGTGGATCATGAGG T-3′ ALU-R, 5′-TTTTTTGAGACGGAGTCTCGC-3′. The hybridization was carried out for 16 h at 42 °C. Signals were detected by immunohistochemistry using anti-digoxigenin alkaline phosphatase-conjugated Fab fragments (Roche Diagnostics GmbH, Mannheim, Germany) and NBT/BCIP (Roche Diagnostics GmbH) as the substrate.

### Human pluripotent stem cell antibody array

Nitrocellulose membranes to capture antibodies were spotted, and reagents for detection were obtained using a kit from R&D Systems (Wiesbaden, Germany). According to the manufacturer's instructions, protein extracts were prepared and incubated with the nitrocellulose membranes, followed by detection of specific protein binding with biotinylated secondary antibodies using streptavidin-HRP and chemiluminescence detection reagents.

### Transplantation of GCTB stromal cells on the CAM of fertilized chicken eggs

This assay was performed as described recently^47^ using white Leghorn fertilized chicken eggs (Geflügelzucht Hockenberger, Eppingen, Germany). The eggs were opened at day 4 of embryonic development, and cells in 50% Matrigel/PBS were transplanted at day 9 on the CAM of eggs with viable embryos. The tumor take and tumor growth were evaluated at day 17. All embryos that died before day 17 were excluded from further analyses. Tumor volumes were estimated using the following formula: volume=4/3 × Π × *r*^3^ (*r*=1/2 × √ of diameter 1 × diameter 2).^33^

### Subcutaneous transplantation of GCTB stromal cells into mice

Adherent (1 × 10^6^) or spheroidal (1 × 10^5^) cultures of GCTB stromal Pat-2, Pat-3 and Pat-8 cells were transplanted in 50% Matrigel/PBS subcutaneously to the left and right flanks of 5-week-old female NMRI-nu immunodeficient mice (Janvier Labs, Saint-Berthevin, France) in a total volume of 100 *μ*l. The tumor engraftment and tumor size were measured and analyzed as described previously.^45^ The animal experiments were carried out in the animal facilities of the University of Heidelberg after approval by the authorities (Regierungspräsidium, Karlsruhe, Germany).

### Statistical analysis

All *in vitro* experiments except the pluripotent stem cell array (Figure 2) and the colony-forming assays (Figures 1c and 5c) were performed in triplicates and the quantitative data are presented as the mean±S.D. The human pluripotent stem cell antibody array was performed with eight different primary cell lines once in duplicate (*n*=2) to get a general overview about stem cell signaling, which did not require statistically significant data. The colony-forming assays were performed twice but in sextuplicates, which ensured a statistically relevant group size (*n*=12). For the *in vivo* transplantation experiments, we used 8 eggs (*n*=8) or 15 mice (*n*=15) per group. The significance of data was analyzed using Student's *t*-test. *P*<0.05 was deemed to be statistically significant.

## Abbreviations

CSC: cancer stem cell
GCTB: giant cell tumor of bone
XL184: cabozantinib
TRAP: tartrate-resistant acid phosphatase
