# Supplementary figures and images for: Enrichment of c-Met+ tumorigenic stromal cells of giant cell tumor of bone and targeting by cabozantinib
Source: Cell Death Dis. 2014 Oct 16;5(10):e1471–. doi: 10.1038/cddis.2014.440 (PMC4237261; doi:10.1038/cddis.2014.440)

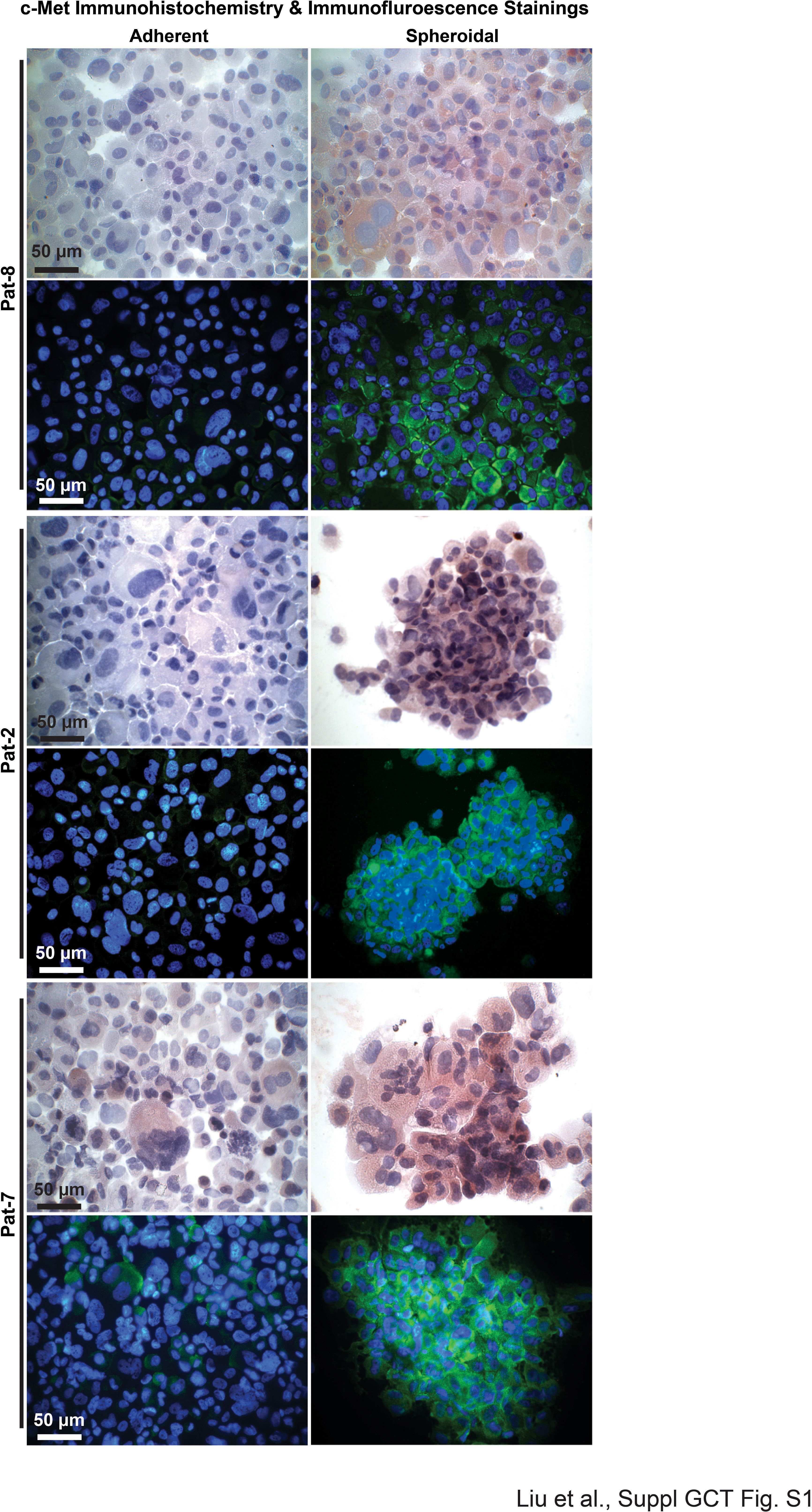

Supplement: Supplementary Figure S1 [file cddis2014440x1.tif]
