# Supplementary material for: Enrichment of c-Met+ tumorigenic stromal cells of giant cell tumor of bone and targeting by cabozantinib
Source: Cell Death Dis. 2014 Oct 16;5(10):e1471–. doi: 10.1038/cddis.2014.440 (PMC4237261; doi:10.1038/cddis.2014.440)
Supplement: Supplementary Table S1 [file cddis2014440x2.docx]

***Table S1.*** ***Characteristics of patients from whose the GCTB species are derived***

| **Name** | **Age in years** | **Gender** | **Tumor Localization** |
| --- | --- | --- | --- |
| Pat-1 | 58 | male | Patella |
| Pat-2 | 34 | female | Distal Femur |
| Pat-3 | 51 | female | Lower Leg |
| Pat-4 | 29 | male | Distal Radius |
| Pat-5 | 28 | female | Tibia Proximal |
| Pat-6 | 16 | female | Distal femur |
| Pat-7 | 60 | male | Humerus |
| Pat-8 | 40 | male | Tibia Proximal |
