# Supplementary material for: Enrichment of c-Met+ tumorigenic stromal cells of giant cell tumor of bone and targeting by cabozantinib
Source: Cell Death Dis. 2014 Oct 16;5(10):e1471–. doi: 10.1038/cddis.2014.440 (PMC4237261; doi:10.1038/cddis.2014.440)
Supplement: Supplementary Table S2 [file cddis2014440x3.docx]

***Table S2. Summary of CSC characteristics in primary GCTB stromal cells***

|  | **CSC Characteristics** | **Pat-**  **1** | **Pat-**  **2** | **Pat-**  **3** | **Pat-**  **4** | **Pat-**  **5** | **Pat-**  **6** | **Pat-**  **7** | **Pat-8** |
| --- | --- | --- | --- | --- | --- | --- | --- | --- | --- |
| Fig. 1B | Spheroids | +++ | +++ | + | + | +++ | ++ | +++ | +++ |
| Fig. 1C | Colonies | ++ | +++ | +++ | + | +++ | ++ | +++ | + |
| Fig. 1D | Differentiation | ++ | +++ | +++ | - | +++ | ++ | - | + |
| Fig. S1A | Migration | +++ | ++ | + | + | ++ | ++ | +++ | + |
| Fig. S1B | CXCR4 | +++ | + | +++ | + | - | + | + | +++ |
| **Summary (+)** | | **13** | **12** | **11** | **4** | **11** | **9** | **10** | **9** |

Absent (-), Weak (+), Median (++), Strong (+++).
