# Supplementary material for: Enrichment of c-Met+ tumorigenic stromal cells of giant cell tumor of bone and targeting by cabozantinib
Source: Cell Death Dis. 2014 Oct 16;5(10):e1471–. doi: 10.1038/cddis.2014.440 (PMC4237261; doi:10.1038/cddis.2014.440)
Supplement: Supplementary Table S3 [file cddis2014440x4.docx]

***Table S3. Human pluripotent stem cell antibody array***

| **Marker** | **Function** | **Ref** |
| --- | --- | --- |
| AFP | α-Fetoprotein, fetal form of serum albumin, tumor marker. | (1) |
| Oct-3/4 | Transcription factor involved in self-renewal. | (2) |
| NANOG | Transcription factor involved in maintaining [pluripotency](http://en.wikipedia.org/wiki/Pluripotency) of stem cells. | (3) |
| SOX2 | Transcription factor essential for the self-renewal and pluripotency. | (4) |
| E-Cadherin | Cell-cell adhesion glycoprotein, loss of function contributes to progression. | (5) |
| GATA-4 | Zinc-finger transcription factor, decreased in carcinogenesis. | (6) |
| FOXA2 | DNA-binding protein, dysregulation in inflammation, tumorigenesis, EMT. | (7, 8) |
| PDX-1 | Transcription factor, reprogramming in mouse pancreas. | (9) |
| SOX17 | Transcription factor, differentiation and antagonization of self-renewal. | (10) |
| Otx2 | Homeobox transcription factor, controls brain morphogenesis + development. | (11) |
| p63 | p53 family, important for development; loss involved in tumorigenesis. | (12) |
| GSC | The goosecoid homeobox gene is repressed during stem cell differentiation. | (13) |
| Snail | Zinc-finger transcription factor involved in EMT, inhibitor E-Cadherin. | (14) |
| VEGF R2 | Main VEGF receptor, angiogenesis, proliferation, migration, survival. | (15) |
| hCG | Peptide hormone that meditates immune tolerance in pregnancy and tumors. | (16) |

**References**

1. Tomasi TB, Jr. Structure and function of alpha-fetoprotein. Annu Rev Med. 1977;28:453-65.

2. Niwa H, Miyazaki J, Smith AG. Quantitative expression of Oct-3/4 defines differentiation, dedifferentiation or self-renewal of ES cells. Nat Genet. 2000;24:372-6.

3. Mitsui K, Tokuzawa Y, Itoh H, Segawa K, Murakami M, Takahashi K, et al. The homeoprotein Nanog is required for maintenance of pluripotency in mouse epiblast and ES cells. Cell. 2003;113:631-42.

4. Niwa H, Ogawa K, Shimosato D, Adachi K. A parallel circuit of LIF signalling pathways maintains pluripotency of mouse ES cells. Nature. 2009;460:118-22.

5. Hazan RB, Qiao R, Keren R, Badano I, Suyama K. Cadherin switch in tumor progression. Ann N Y Acad Sci. 2004;1014:155-63.

6. Akiyama Y, Watkins N, Suzuki H, Jair KW, van Engeland M, Esteller M, et al. GATA-4 and GATA-5 transcription factor genes and potential downstream antitumor target genes are epigenetically silenced in colorectal and gastric cancer. Mol Cell Biol. 2003;23:8429-39.

7. Liu M, Lee DF, Chen CT, Yen CJ, Li LY, Lee HJ, et al. IKKalpha activation of NOTCH links tumorigenesis via FOXA2 suppression. Mol Cell. 2012;45:171-84.

8. Song Y, Washington MK, Crawford HC. Loss of FOXA1/2 is essential for the epithelial-to-mesenchymal transition in pancreatic cancer. Cancer Res. 2010;70:2115-25.

9. Zhou Q, Brown J, Kanarek A, Rajagopal J, Melton DA. In vivo reprogramming of adult pancreatic exocrine cells to beta-cells. Nature. 2008;455:627-32.

10. Niakan KK, Ji H, Maehr R, Vokes SA, Rodolfa KT, Sherwood RI, et al. Sox17 promotes differentiation in mouse embryonic stem cells by directly regulating extraembryonic gene expression and indirectly antagonizing self-renewal. Genes Dev. 2010;24:312-26.

11. Bai RY, Staedtke V, Lidov HG, Eberhart CG, Riggins GJ. OTX2 represses myogenic and neuronal differentiation in medulloblastoma cells. Cancer Res. 2012;72:5988-6001.

12. Flores ER, Lozano G. The p53 family grows old. Genes Dev. 2012;26:1997-2000.

13. Kalisz M, Winzi M, Bisgaard HC, Serup P. EVEN-SKIPPED HOMEOBOX 1 controls human ES cell differentiation by directly repressing GOOSECOID expression. Dev Biol. 2012;362:94-103.

14. Davidson NE, Sukumar S. Of Snail, mice, and women. Cancer Cell. 2005;8:173-4.

15. Holmes DI, Zachary IC. Vascular endothelial growth factor regulates stanniocalcin-1 expression via neuropilin-1-dependent regulation of KDR and synergism with fibroblast growth factor-2. Cell Signal. 2008;20:569-79.

16. Triozzi PL, Stevens VC. Human chorionic gonadotropin as a target for cancer vaccines. Oncol Rep. 1999;6:7-17.
