# Supplementary material for: Enrichment of c-Met+ tumorigenic stromal cells of giant cell tumor of bone and targeting by cabozantinib
Source: Cell Death Dis. 2014 Oct 16;5(10):e1471–. doi: 10.1038/cddis.2014.440 (PMC4237261; doi:10.1038/cddis.2014.440)
Supplement: Supplementary Table S4 [file cddis2014440x5.docx]

***Table S4. Induction of stem cell markers in spheroidal versus adherent cells***

| **Stem Cell Marker** | **Patient No.** | | | | | | | |
| --- | --- | --- | --- | --- | --- | --- | --- | --- |
|  | Pat-1 | Pat-2 | Pat-3 | Pat-4 | Pat-5 | Pat-6 | Pat-7 | Pat-8 |
| Oct-3/4 | - | ++ | + | +++ | + | + | + | +++ |
| Nanog | + | +++ | + | +++ | - | - | - | - |
| SOX2 | - | ++ | - | + | - | - | - | + |
| E-Cadherin | - | + | + | + | - | - | - | - |
| α-Fetoprotein (AFP) | - | + | +++ | +++ | ++ | - | +++ | ++ |
| GATA-4 | + | + | + | + | - | - | - | ++ |
| HNF-3β/FoxA2 | +++ | +++ | +++ | +++ | - | ++ | ++ | - |
| PDX-1/IPF1 | ++ | + | ++ | ++ | - | - | - | - |
| SOX17 | - | + | + | ++ | ++ | - | - | +++ |
| Otx2 | + | - | - | + | - | - | - | +++ |
| TP63/TP73L | + | - | - | - | - | - | ++ | - |
| Goosecoid (GSC) | ++ | - | + | + | - | - | - | - |
| Snail | + | - | - | + | ++ | - | - | ++ |
| VEGF R2/KDR/Flk-1 | +++ | + | +++ | + | - | - | - | ++ |
| HCG | ++ | - | - | - | - | - | + | - |
| **Summary (+)** | **17** | **16** | **17** | **23** | **7** | **3** | **9** | **18** |

Grading of induction in Fig. 3: <10 % (-), 10-50 % (+), 50-99 % (++), >100 % (+++).

Grading of summary for Table 2: > 17 = +++; > 8 = ++; > 3 = +.
